# Metal Alloys Used in Dental Prosthetics and Their Impact on the Oral Microbiome: Narrative Review

**DOI:** 10.3390/dj14080506

**Published:** 2026-08-10

**Authors:** Iwona Ordyniec-Kwaśnica, Anna Kudra, Mateusz Lampkowski, Damian Muszyński

**Affiliations:** 1 Division of Dental Prosthetics, Medical University of Gdansk, 80-208 Gdansk, Poland; mateusz.lampkowski@gumed.edu.pl; 2 “Zdrowie” LLC DENTUS Dental Clinics in Gdynia, 81-364 Gdynia, Poland; annakudra@gumed.edu.pl; 3 Pomeranian Dental Center Kryspin-Dent, 80-288 Gdansk, Poland

**Keywords:** dental prosthetics, metal alloys, oral microbiome, dentistry, prosthetic crowns, implantology

## Abstract

**Introduction**: One of the fundamental principles of modern medicine, including dentistry, is prevention. However, if treatment is not initiated, tooth loss can occur, necessitating the use of dental prostheses to restore the function and aesthetics of the stomatognathic system. The oral microbiome is a complex ecosystem that is sensitive to external factors, including the biomaterials used to manufacture dental prostheses. **Objectives**: This narrative literature review aims to identify and compare the effects of various metal alloys used in dentistry, specifically high-precious (gold), precious (silver–palladium) and base (cobalt–chromium and nickel–chromium) alloys, as well as titanium, on the balance of the oral microbiome and biofilm formation. **Results**: The analysis indicates that gold, silver and palladium alloys demonstrate the most favourable biocompatibility and antibacterial properties, significantly reducing biofilm accumulation. Titanium and titanium-based alloys generally exhibit neutral properties under healthy conditions, although their biocorrosion products can alter the microbial environment in pathological states such as peri-implantitis. In contrast, base metal alloys (cobalt–chromium and nickel–chromium) are highly susceptible to biocorrosion in acidic environments, which can encourage the growth of bacteria that cause tooth decay and inflammation, potentially exacerbating oral dysbiosis. **Conclusions**: The selection of materials plays a critical role in maintaining oral microbial homeostasis and preventing plaque-related diseases. High-noble alloys and titanium are more biocompatible than base metal alloys. However, further long-term clinical trials and multi-species biofilm models are needed to fully understand these interactions between materials and microbes.

## 1. Introduction

One of the most important roles of doctors and dentists is to restore patients’ mental and physical health. Dental prosthetics fulfil this role by restoring proper function and aesthetics to patients with qualitative (understood as restorations of a portion of the tooth in the form of a crown or an inlay, designed to restore its original function) and quantitative deficiencies. This is achieved through the use of appropriate prostheses, which can be removable or fixed. Due to their constant contact with the complex environment of the oral cavity, denture materials must meet several guidelines [1,2]. The most important guidelines for metal alloys are that they cause no allergies or toxicity, are low in corrosion susceptibility in the oral cavity, and have tensile strength, hardness, and durability under chewing loads. Currently, these guidelines are met by several different alloys. These include gold alloys, silver–palladium alloys, cobalt-chrome alloys and nickel–chromium alloys. These metal alloys can be divided into three groups: high-noble alloys; noble alloys; and non-precious alloys. The first group includes metal alloys characterised by a composition of at least 60% gold and high corrosion resistance. These are mainly used for metal-ceramic or metal restorations (e.g., gold alloys). Precious alloys, on the other hand, contain at least 25% precious metals and have similar corrosion resistance to high-precious alloys (e.g., silver–palladium alloys). Base alloys contain up to 25% noble metals and are used to make bridges, crowns, and partial dentures [3]. The varied composition of alloys used in dental prosthetics affects their physical and biological properties. The presence of specific elements may play a key role in corrosion resistance [4]. The oral microbiome, which constitutes a dynamic ecosystem, is similarly complex. It is characterised by the presence of various bacteria, viruses, and archaea that can interact with each other and with factors in the surrounding environment, thereby affecting the microbiological balance [5]. A similar situation can be observed with metal alloys, where the selection of elements can alter the alloy’s properties [4].

Although the mechanical performance and electrochemical properties of noble and base metal dental alloys are well established, their long-term impact on the oral ecosystem is a critical area of ongoing research. While current literature focuses heavily on material stability and single-species bacterial adhesion, primarily targeting *Streptococcus mutans* [6,7,8], a significant gap remains in our understanding of how different alloy compositions affect multi-species biofilms and oral homeostasis in vivo [9,10,11]. Furthermore, the precise role of chronic biocorrosion and metal ion release in promoting oral dysbiosis, i.e., shifting the balance from commensal bacteria to periodontal pathogens, is still poorly understood [12,13,14]. Therefore, an in-depth synthesis of the current state of knowledge is essential to understand how these materials interact with the complex oral environment.

The similarity between metal alloys and the microbial environment of the oral cavity means they can influence each other. The primary function of dental prostheses is to rehabilitate the stomatognathic system by restoring lost functions, including lost teeth [3]. Therefore, this rehabilitation may also extend to the oral microbiome through the antibacterial or stabilising effects of certain metal alloys on the microbiome. Accordingly, the terms “rehabilitation” and “positive factors” will be used interchangeably in this publication.

## 2. Methodology

### 2.1. Literature Search Strategy and Framework

To ensure a structured, transparent and high-quality presentation of the literature, this review was prepared in accordance with the Scale for the Assessment of Narrative Review Articles (SANRA) guidelines. A comprehensive literature search was performed across electronic databases, including PubMed/MEDLINE, Scopus, and Web of Science, covering the period from 1990 to 2025. The search strategy was based on a combination of medical subject headings (MeSH) and relevant keywords such as: dental alloys, gold alloy, silver–palladium alloy, cobalt–chromium alloy, nickel–chromium alloy, titanium alloys, oral microbiome, biofilm, dental plaque, corrosion, biocorrosion, metal ion release, *Streptococcus mutans*, periodontal bacteria, biofilm formation. Additionally, the references cited in selected articles were analysed to identify further relevant publications. The review included publications from 1990 to 2025, with a particular focus on fundamental research on corrosion and microbiology.

### 2.2. Selection Criteria

The following criteria were adopted for literature selection:

Inclusion criteria:

→ Studies analysing interactions between dental alloys and microorganisms;
→ Studies on corrosion, metal ion release, or biofilm formation;
→ In vitro, in vivo, and clinical studies;
→ Review articles containing an analysis of mechanisms

Exclusion criteria:

→ Studies unrelated to dental prosthetics (e.g., orthopaedic implants with no mechanistic relevance);
→ Studies with no reference to microbiology or material properties;
→ Case reports with no mechanistic relevance;
→ Sources not subject to peer review were strictly excluded

### 2.3. Structural Quality Assessment

To enhance the coherence and internal validity of this narrative review, all included studies were critically evaluated based on the principles of the SANRA framework. Specifically, the literature was appraised regarding the clarity of its scientific rationale, the precision of its endpoints, and the robustness of its experimental models (in vitro vs. in vivo), ensuring that the synthesis of material-microbiome interactions remains grounded in high-quality evidence.

## 3. Results and Discussion

### 3.1. Oral Healthy Microbiome

**Figure 1:**
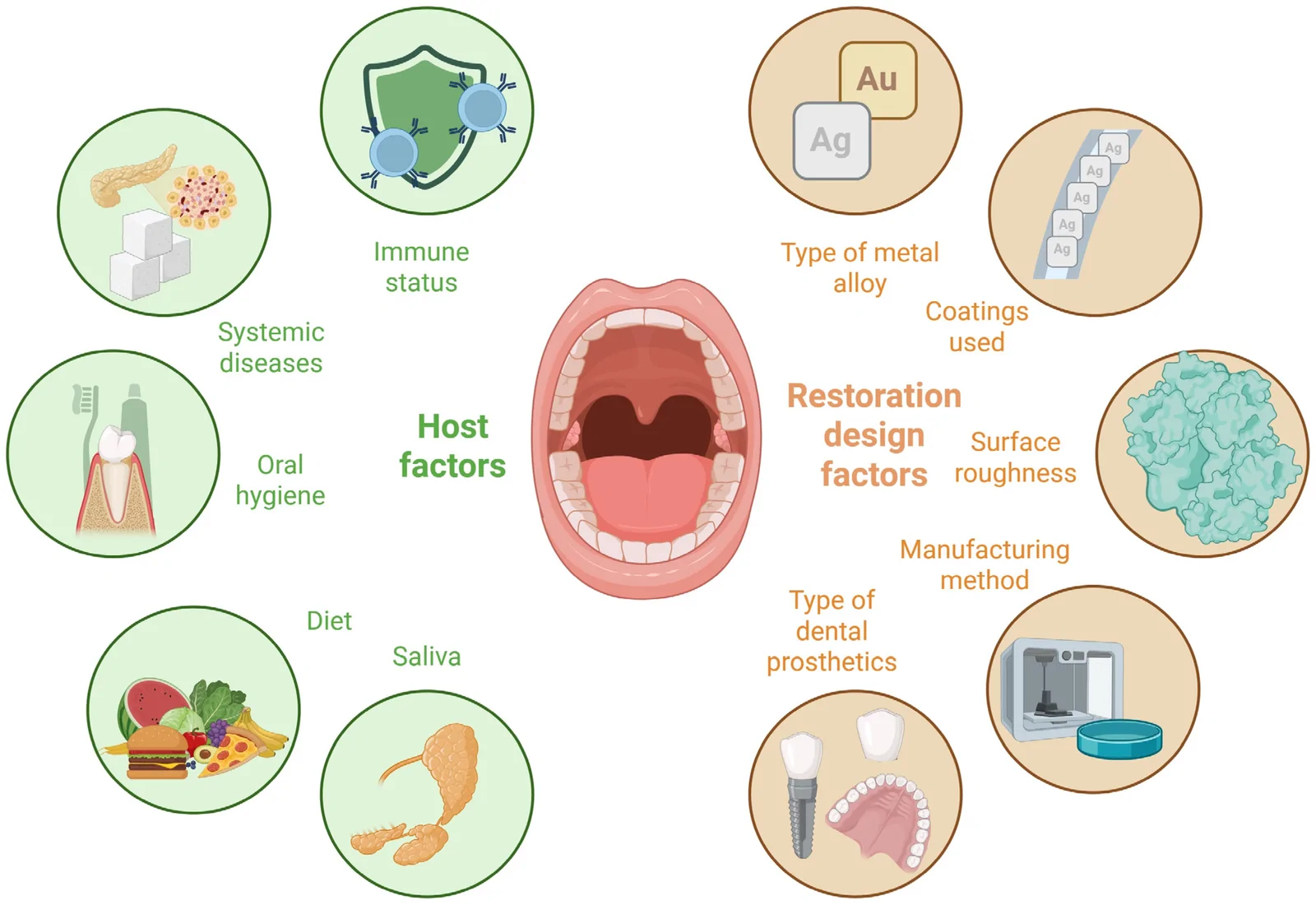
An overview of the most important factors influencing the oral microbiome from the perspective of dental prosthetics. Created with BioRender.com.

The oral microbiome is a complex and diverse ecosystem that is highly sensitive to internal and external factors. Its dynamic variability is closely related to human immune status, and it also acts as a form of communication with other organs in the body. From the perspective of dental prosthetics, significant changes may occur as a result of both host factors and factors related to the design of the prosthetic restoration itself, as shown in Figure 1, which presents an overview of the potential influence of common dental metal alloys on the oral microbiome. Biocompatibility, antimicrobial characteristics, and susceptibility to biocorrosion are all important factors. As presented, gold alloys appear to have the most favourable profile, as well as silver–palladium alloys, although the current information is sparse and inconsistent. Titanium and titanium-based alloys show no significant impact, whereas base metal alloys, particularly cobalt–chromium and nickel-chromium, show limited or no beneficial impact on oral health. Changes to it may indicate the onset of certain diseases, such as cancer or diabetes. It is estimated that over 700 species of bacteria inhabit the oral cavity, comprising its microbiome [15,16]. A thorough analysis of 16S DNA in a healthy oral cavity enabled the classification of a wide variety of bacterial groups, including *Bacillota* (formerly *Firmicutes*), *Actinomycetota* (formerly *Actinobacteria*), *Pseudomonadota* (formerly *Proteobacteria*), *Fusobacteria*, *Bacteroidota* (formerly *Bacteroidetes*), and *Spirochaetes*. The bacteria mentioned above make up 96% of the oral microbiome [16].

*Bacillota* (formerly *Firmicutes*) are a type of Gram-positive bacterium that includes *Lactobacillus* spp. and *Streptococcus* spp. [17]. *Lactobacilli* are bacteria closely associated with dental caries. They are characterised by acidogenicity and acid resistance, as well as the ability to bind to collagen and form extracellular polysaccharides. These features make them ideal for enabling the growth of bacterial plaque. However, the growth of this plaque may be limited to *Lactobacillus* only, as it is able to inhibit the growth of other cariogenic bacteria due to competition [18]. *Streptococcus* bacteria are often referred to as the primary colonisers of the oral cavity and therefore play an important role in forming the oral microbiome. One of the most recognisable representatives of this group is *Streptococcus mutans*, which is closely related to the development of dental caries [19]. Its metabolism is mainly based on organic acids, thus acidifying the local environment [20]. Other bacteria in this genus, such as *Streptococcus salivarius*, produce large amounts of bases, which play an important role in controlling the acid–base balance [19]. Another important member of the *Bacillota* is *Streptococcus dentisani*. This bacterium has the ability to buffer acidic pH levels and also inhibits the growth of pathogens present in the oral cavity. For these reasons, it is often presented as a probiotic against dental caries [21].

*Actinomyces* is a genus of Gram-positive bacteria that is commonly found on oral mucosal surfaces [22,23]. Types such as *Actinomyces isrealii* are well-known bacteria that cause *actinomycosis*. They do not cause any harmful changes in healthy individuals. In contrast, trauma, surgery, or a decreased immune response may cause them to become dangerous pathogens in the human body [22]. *A. isrealii* can multiply in the human body following certain infections, such as those caused by the SARS-CoV-2 virus. Therefore, it is important to make an accurate diagnosis in order to initiate appropriate treatment, given that the incidence of *actinomycosis* in patients with SARS-CoV-2 is significantly lower than that of fungal infections [24]. The pathogenicity of *Actinomyces* species is also attributed to their fimbriae. These are present, for example, in *Actinomyces oris*, which influences the potential interaction between them and streptococci, which serve as a bridge for the development of dental plaque. This means that the presence of *Actinomyces oris* may potentially increase the proportion of streptococci in plaque formation, thereby increasing the risk of caries development. Research by Mishra et al. has shown that the adhesive activity of FimA, a component of fimbrin, plays a significant role in the adhesion capacity of *A. oris*, which could be utilised in the future to control plaque formation [25]. The function of an interspecies bridge is also confirmed by a study conducted by Dige et al., which demonstrated that *Actinomyces naeslundii* is located in the inner regions of the biofilm, indicating that it serves as the attachment site for subsequent components of the future multi-species biofilm [26].

*Proteobacteria* (now *Pseudomonadota*) are one of the largest groups of Gram-negative bacteria and belong to the so-called ‘iron bacteria’. Depending on environmental conditions, they have the ability to oxidise or reduce iron [27]. Nevertheless, they are not commonly found in the oral cavity; the most notable representatives are the *Neisseriaceae*, *Pasteurellaceae* and *Campylobacteraceae* families. Growth of the *Pseudomonadota* is often associated with inflammatory diseases [28]. From a dental perspective, the most notable example of this group is *Aggregatibacter actinomycetemcomitans*. This bacterium is often found alongside *Actinomyces* and is responsible for severe periodontitis [29].

*Fusobacteria* are anaerobic, Gram-negative bacteria. The most important species is *Fusobacterium nucleatum*. This bacterium is characterised by its association with various systemic diseases, including vascular and rheumatological diseases, as well as oral disorders, specifically periodontal and gingival diseases [30]. *F. nucleatum* can bind to and attack epithelial and endothelial cells thanks to its FadA adhesins. Furthermore, an excess of *F. nucleatum* in pregnant women can lead to complications such as neonatal sepsis or stillbirth [30].

*Bacteroidota* (formerly *Bacteroidetes*) are Gram-negative bacilli that occur in increased numbers in people with diabetes and periodontal disease. In the case of dental caries in children, however, the opposite situation occurs, with a reduced share of *Bacteroidota* [31]. Representative of this group, *Porphyromonas gingivalis*, is also responsible for diseases affecting the periodontium and gums. Due to its high virulence, *P. gingivalis* is classified as a key member of the ‘red complex’. This bacterium can transfer to a large number of cells, which correlates with changes in hair growth, while bypassing the host’s defence mechanisms [32]. Another important member of the *Bacteroidota* is *Tannarella forysthia*, which is also a member of the “red complex.” It is a late coloniser of the oral cavity, with virulence factors such as sialidase and protease, which define the pathogenicity of *T. forysthia*. *T. forysthia* contributes to the development of periodontal tissue disorders [33].

*Treponema denticola* is a *Spirochete* strongly associated with periodontitis and is considered a key member of the “red complex”, a group of bacteria linked to advanced periodontal disease [34,35]. Periodontal diseases are among the most prevalent oral conditions globally, affecting a large proportion of adults in their mild forms.

### 3.2. Mechanisms of Biocorrosion and Tribocorrosion

As previously mentioned, materials commonly used in dental prosthetics must meet many criteria. Due to being placed in the complex environment of the oral cavity, they are constantly exposed to various factors, such as saliva, the microbiome, food, and temperature fluctuations. All of these factors may influence the development of corrosion. Corrosion is defined as the gradual destruction of metal resulting from interaction with various factors. Due to the complexity of the concept of ‘various factors’, there are many potential causes of corrosion. They may depend on the type of metal alloy used, the process by which the alloy was obtained, the type of laboratory processing, and oral hygiene [36].

The presence of a foreign body in the mouth creates conditions conducive to the formation of bacterial biofilms. The bacteria contained in these biofilms contribute to corrosion [36]. One of the most prevalent bacteria in the oral cavity is *Streptococcus mutans*, which can colonise the oral cavity and form biofilms [37]. Souza’s research on the presence of *Streptococcus mutans* suggests that it may impact the corrosion of alloys [6]. The activity of cariogenic bacteria ultimately leads to the formation of acids that directly react with the metal, causing corrosion [36,38]. As corrosion develops, corrosion cells may form. These form when two adjacent areas come into contact with solutions of different concentrations. There are three types of cells. Cells with different degrees of oxygenation arise between areas with different oxygen concentrations. The second type of cell contains different concentrations of metal ions and is formed due to differences in metal ion concentration. Passive-active cells are created on similar principles to the first two types [36,39].

Understanding the corrosion process is important for determining the subsequent impact of individual metal alloys on the oral microbiome. As a result of corrosion, acids are produced [38]. A similar relationship occurs in the growth of *S. mutans*, for example. The metabolism of *S. mutans* leads to the production of organic acids, primarily lactic acid. Environmental acidification creates ideal conditions for the growth of *S. mutans* [20]. Therefore, since the corrosion process also produces acids, the causative bacterial agents may be able to multiply, affecting the balance of the oral microbiome.

In addition to conventional corrosion, dental alloys are also susceptible to tribocorrosion, a dynamic process that combines two fields—tribology and corrosion—and can occur in the oral cavity [40]. This occurs because, when a dental implant, for example, is placed in the oral cavity, the material from which it is made is constantly exposed to biological fluids while simultaneously undergoing mechanical stress. Repeated chewing and acidification caused by biofilm can compromise protective oxide layers, thereby increasing ion release and altering the local microbial environment. Recent reports on biomaterials indicate that the rate of oxide layer regeneration has a decisive impact on resistance to tribocorrosion, and the process itself is (as the name suggests) quite dynamic. The oral environment itself also plays a significant role here. A good example of this is saliva, which affects the pH of the oral cavity and its constituent elements. The presence of citric acid under experimental conditions, for example, has an ambiguous effect on the phenomenon of biotribocorrosion. Interestingly, the presence of fluoride may, for example, have a negative effect on resistance to biotribocorrosion [6,9,41].

### 3.3. Materials in Dental Prosthetics and Impact on the Oral Microbiome

Currently, there is a wide variety of materials used in dental prosthetics for the fabrication of crowns, inlays, and onlays, which may have some impact on the surrounding environment. First, a biofilm forms on the metal surface, determining the composition of the microbiome [42]. The growing biofilm can lead to the previously described corrosion and the release of metal ions [43]. The following section presents descriptions of the most commonly used alloys, their properties, and their potential impact on the oral microbiome.

#### 3.3.1. Gold Alloy

A. Material characteristics.

Gold is a type of light yellow precious metal with very good chemical resistance. Its utilisation in dentistry commenced in 1932 [44]. However, some sources claim that it has been used for up to 4000 years [10]. Gold’s optimal prosthetic properties, including its remarkable malleability and softness, have led to its longstanding utilisation as the preferred material in dentistry. One of the most significant methodologies employed in the acquisition of gold-based artefacts is electroforming, a technique that facilitates the precise fabrication of prosthetic restorations devoid of any artefacts [44]. Nevertheless, gold is most often utilised in the form of alloys, a consequence of the material’s inherent disadvantages, namely its high ductility and the associated expense. The combination of gold with other materials has been demonstrated to reduce the financial outlay associated with prosthetic work whilst simultaneously enhancing its durability [45].

B. Correlation with the oral microbiome.

⮚ Direct evidence

Regarding direct clinical and laboratory evidence, gold is characterised by a high degree of biocompatibility. This claim is supported by the results of a comparative study conducted by Goodson et al. A total of nine patients (in vivo) with healthy periodontal dentition were selected for the collection of bacterial samples from the oral cavity. A total of 42 gold-containing crowns were identified in these same patients. This approach allows for a partial replication of biological conditions. Nevertheless, the small sample size and the nature of the experiment (in vitro/ex vivo) limit the ability to generalise the results to the clinical population. The number of individual bacteria in the samples was determined using the DNA:DNA hybridization method. The results showed that the surfaces of gold crowns exhibited a significantly lower number of bacteria compared to the surfaces of control samples (by approximately 71%). It is worth noting that the percentage composition did not show significant differences between the groups [46]. The rationale behind this phenomenon is that gold, in conjunction with other metal oxides, exhibits peroxidase-like activity. These molecules possess the capacity to degrade H_2_O_2_ at low pH values, a process which can result in the degradation of biofilms and the subsequent destruction of embedded bacteria. Gold and its alloys exhibit the ability to self-passivate. These conclusions were reached by Capopreso et al. In the cited study, more than a dozen different dental alloys, including gold, were examined; they were analysed before and after polishing, as well as after exposure to corrosive conditions. Bacterial adhesion and growth were also tested. Gold, like other alloys, showed a tendency toward passivation. However, it is worth noting that gold had the lowest electrochemical potential, which meant it performed best in terms of corrosion resistance [47].

⮚ Circumstantial evidence

In contrast, indirect or circumstantial evidence in the literature often relies on different material scales and general microbiological models. For instance, it is noteworthy too that metal nanoparticles have the capacity to demonstrate the protective properties of hydroxyapatite, a property that is advantageous in the treatment of caries, particularly that caused by *Streptococcus mutans*, the predominant bacterium responsible for carious lesions in the oral cavity [7,48]. In the case of gold and its alloys, research focuses mainly on the aetiology of dental caries. Apart from *S. mutans*, other causative agents include, but are not limited to, *Lactobacillus* spp., *S. mitis* and *S. sorbinus* [49]. In the study by Tandon et al., the statistical analysis of *Lactobacillus* spp. and *S. mutans* in children demonstrated that the bacterial colonies exhibited comparable levels of curativeness [50]. This finding may imply that *Lactobacillus* plays a comparable significant role in the development of carious lesions. The hypothesis that gold exerts a reducing effect on bacterial plaque may suggest a simultaneous effect on other cariogenic bacteria; however, the extent to which it affects individual bacterial groups is not yet known. It is also important to note that gold and its alloys have been shown to reduce the amount of bacterial plaque, but do not alter the proportion of bacteria present. This finding indicates that the presence of gold does not appear to have a significant impact on the risk of developing plaque-related diseases, as it does not appear to promote or reduce the growth of specific bacterial strains [46].

C. Limitations and conclusions

The extant literature does not provide any studies that definitively demonstrate the impact of gold alloys on the reduction in the risk of periodontal changes. The hypothesis that gold alloys do not promote the development of carious and periodontal lesions is supported by the evidence that they do not do so if they are properly cleaned and oral hygiene is at an appropriate level. This procedure facilitates the long-term maintenance of prosthetic works made of gold within the oral cavity [51]. Nevertheless, any inlay, onlay, or prosthetic crown that has not been properly polished may exhibit rough edges, which may encourage the accumulation of dental plaque and the development of biofilm [52].

The mechanism by which gold interacts with the oral microbiome depends on its corrosion resistance—the higher the resistance, the lower the release of ions. Therefore, high-purity gold alloys reduce bacterial counts by up to 70%, thereby fulfilling their role in “rehabilitating” (i.e., positive changes) the microbiome by reducing bacterial levels. However, most studies involving gold in this category are in vitro or short-term, which may somewhat distort our understanding of this material [53].

#### 3.3.2. Silver–Palladium Alloy

A. Material characteristics.

Silver–palladium alloys, which were introduced around 1973, are now widely regarded as an alternative to prosthetic works made of gold [54,55]. Silver–palladium alloys are characterised by a whitish varnish and are resistant to corrosion (although this property is ambiguous) [56]. Nonetheless, as a result of the casting process used to obtain prosthetic works in dentistry, carbon absorption may have a negative effect on the properties of these alloys. Carbon reduces the mechanical properties of these alloys; therefore, during the casting process, environments devoid of this element should be used [55]. Moreover, a large number of different factors play a role in traditional foundry. These include the type of factors to be considered are alloy, heat source, time and casting method. Consequently, novel methodologies are currently being sought to obtain silver–palladium alloys, for example, vacuum casting in an argon arc [57].

B. Correlation with the oral microbiome.

⮚ Direct evidence

When discussing silver–palladium restorations, a clear distinction must be made between the well-documented properties of silver derivatives and the lack of direct evidence regarding solid prosthetic components. Currently, dedicated clinical or laboratory studies evaluating the direct impact of *bulk* silver–palladium dental alloys on mature, multi-species oral biofilms remain practically unavailable. Consequently, whether the antimicrobial efficiency observed in other silver modalities directly occurs in bulk Ag-Pd prosthetic frameworks remains largely speculative.

⮚ Circumstantial evidence

Silver has been shown to possess antibacterial properties [58]. This phenomenon is attributable to the presence of positively charged ions, which have been demonstrated to induce perturbations within the cell membrane [48]. The utilisation of metallic coatings, encompassing silver, for the surface coating of dental implants has been shown to impede the progression of perimplantitis [58]. Admittedly, this property does not apply to the prosthetic materials themselves, but only to the coating on the material; nevertheless, it is also worth noting that peri-implant inflammations are predominantly associated with Socransky’s red complex, comprising *P. gingivalis*, *T. denticola* and *T. forsythia* [14,59]. For this reason, one could also propose the hypothesis that the utilisation of coatings composed of this particular type of material serves to diminish the propagation of these bacteria through antiseptic mechanisms. As previously mentioned, *P. gingivalis* has been shown to disrupt the equilibrium within a specific ecological niche, thereby contributing to the development of periodontal diseases. Other bacteria belonging to the red complex have been observed to exert a comparable effect, thus promoting the progression of periodontal diseases [59]. The number of bacteria associated with periodontal disease is higher, as mentioned in the previous chapter. It is possible that prosthetic restorations made of silver also have a similar effect in reducing the number of bacteria belonging, among others, to the Socransky red complex.

It has been hypothesised that low concentrations of silver ions may exert an antibacterial effect on *S. mutans* through the mechanism of contact killing of metal nanoparticles. As posited by Zhu et al., silver has the capacity to induce alterations in the proximate environment through the generation of reactive oxygen species (ROS) under conducive conditions. This phenomenon has found application in photothermal therapy [48]. The antibacterial properties of silver were the subject of a systematic review by Hernández-Venegas et al. The conclusions noted that silver (particularly SDF) exhibits strong inhibitory effects against *S. mutans* (but not only), among others, and also reduces biofilm formation and bacterial adhesion to tooth surfaces [60]. Other sources report that silver-based biomaterials are capable of counteracting a significantly larger number of bacteria, including *S. oralis*, *S. sanuginis*, *P. gingivalis*, *S. aureus*, and even *C. albicans* [61]. Whether a similar antimicrobial effect occurs in bulk silver–palladium alloys remains speculative, as dedicated studies in mature oral biofilms are currently unavailable.

C. Limitations and conclusions

Finally, it is worth mentioning the mechanism by which silver–palladium alloys interact. Silver ions are capable of damaging cell membranes, denaturing proteins, or destroying DNA, which ultimately demonstrates that these alloys have antimicrobial properties; in light of these capabilities, they can also be classified as metal alloys that have a beneficial effect on the microbiome as a whole, consequently possibly including the oral microbiome [60]. A significant gap in our understanding of this alloy’s effects is the lack of information and detailed studies on the action of Ag in the oral biofilm. Palladium itself is also controversial due to its theoretical lack of biocompatibility [62].

#### 3.3.3. Cobalt–Chromium Alloy

A. Material characteristics.

Chrome–cobalt alloys are classified as a type of base metal alloys. The popularity of these materials increased in the field of dental prosthetics during the 1930s. As proposed by Al Jabbari et al., the utilisation of these materials is driven by numerous advantages, including but not limited to: reduced heat conduction, enhanced strength, resistance to discolouration, ease of moulding, and minimal shrinkage. These materials are utilised in the fabrication of elements of skeletal dentures and metal-porcelain restorations that are permanently cemented within the patient’s oral cavity. The fundamental method for the production of specific dental prosthetic works is casting [63,64]. In recent years, there has been an increasing use of CAD-CAM methods and selective laser sintering (SLM) [63]. SLM technology offers numerous advantages over traditional casting techniques. A study by Koutsoukis et al. demonstrated that Selective Laser Melting (SLM) technology facilitates more rapid and cost-effective production of prosthetics when compared with casting or milling methods [65].

B. Correlation with the oral microbiome.

⮚ Direct evidence

Pitting corrosion caused by oral biofilm is a phenomenon frequently observed in the presence of nickel and chromium. A particular role is attributed to Streptococcus bacteria [64]. However, it is important to note that Co-Cr alloys exhibit high corrosion resistance [63,65]. Lucchentti et al.’s research sought to ascertain the release of various ions from Co-Cr alloys following interaction with oral bacteria at varying pH levels. The release of elements observed in the results suggested an increase in the number of acidophilic and acid-producing bacteria. However, the magnitude of this increase was minimal, with the exception of the melt in an acidic medium, which has been shown to be particularly conducive to the proliferation of cariogenic bacteria [12]. Consistent with these findings, similar results were reported in the studies conducted by Leban et al. [66]. However, it is worth noting that the focus of this study was to compare Co-Cr alloys produced using two methods: milling and 3D printing. While the study mentions acidic conditions, this is not its primary focus. Results from both studies suggest a potential link between an increased number of cariogenic bacteria and the heightened susceptibility of Co-Cr alloys to corrosion in an acidic environment.

⮚ Circumstantial evidence

The risk of potential biofilm formation can be reduced, for example, by adding copper to the cobalt–chromium alloy, as confirmed by a study conducted by Ren et al. [4]. The study aimed to develop a new metal alloy for dental applications. Among the key requirements listed were, among others, the ability to kill bacteria and prevent biofilm formation. To this end, they produced a CoCrCu alloy using 3D printing and tested it for various aspects, including antibacterial activity, biofilm formation, and material structure. The results indicated that this type of alloy does indeed have the ability to kill bacteria while maintaining biocompatibility [4].

C. Limitations and conclusions

As previously mentioned, chrome–cobalt alloys are utilised in the fabrication of permanent dental prosthetics [63,67]. The application of these alloys is predominantly in the domain of metal-ceramic dentures, a field extensively documented for its association with elevated bacterial levels. As posited by Alarcón-Sánchez et al., an escalation in pro-inflammatory cytokines has the potential to result in the degradation of the supporting tissue of the tooth, consequently precipitating an inflammatory response [67]. Consequently, metal-ceramic dentures may, in an indirect manner, contribute to an augmentation in the population of bacteria responsible for inducing inflammation. However, the current literature is heavily limited by a lack of standardised quantitative metrics, making it challenging to establish a direct causal link between Co-Cr degradation products and specific shifts in the peri-implant microbiome.

In summary, Co-Cr alloys are associated with a higher risk of corrosion than the metal alloys described earlier. It is true that they have a Cr_2_O_3_ layer, which increases corrosion resistance [68]. However, the production of acids by microorganisms in the oral cavity leads to the destabilisation of the oxide layer [64]. It is true that the specific characteristics of the microbiome for Co-Cr alloys have not yet been fully elucidated, nor has the impact of various processing methods on this material. It is therefore not possible to definitively determine the positive impact of this alloy.

#### 3.3.4. Nickel–Chromium Alloy

A. Material characteristics.

The introduction of dental prosthetics made of this alloy dates back to the 1930s. Nonetheless, there has been a gradual shift away from nickel–chromium alloys in dental prosthetics. This shift is due to the introduction of improved chrome–cobalt alloys, which are increasingly replacing nickel–chromium alloys due to their numerous disadvantages, including high shrinkage during casting and the potential allergic and toxic effects of Ni. As posited by Al Jabbari et al., the allergic effect is predicated on the release of metal ions [63]. Arakelyan et al. posit that these ions are gradually released under the influence of various local or general factors and then accumulate in the oral mucosa. If a patient is allergic to a given group of metal ions, they may experience a type IV hypersensitivity reaction, which mainly affects the skin and mucous membrane. As posited by Arakelyan et al., in cases of greater complexity, there is the potential for exacerbation of other diseases, including autoimmune diseases that affect the skin, salivary glands, or joints [13].

B. Correlation with the oral microbiome.

⮚ Direct evidence

The toxic effect of nickel (Ni) is attributable to its capacity to bind directly or indirectly with oxygen (O), nitrogen (N), and sulphur (S) atoms, or with negatively charged ions within the body. It has been demonstrated that oral biofilm can significantly accelerate the dissolution of Ni^2+^ ions, thereby initiating the process of pitting corrosion. It can be concluded that the potential risk of caries may also increase due to the subsequent proliferation of cariogenic bacteria that are responsible for the occurrence of caries in the oral cavity [64]. This theory is supported, for instance, by Gopalakrishnan’s review, which reports a correlation between biofilm and the corrosion of chromium-nickel alloys [43]. Furthermore, Xiong et al. observed that *Actinomyces viscosus* can also induce alloy corrosion. In the study conducted by Laurent et al., this Gram-positive bacterium was identified as the causative agent of the corrosion of the chromium-nickel alloy. This bacterium has been shown to be associated with caries, typically located on the root surface of teeth, often in conjunction with *Candida albicans* [69,70].

⮚ Circumstantial evidence

The action of *Streptococcus* spp. bacteria (in particular *S. sanguinis* and *S. mutans*) has been demonstrated to cause an increased amount of release of these ions in the case of orthodontic archwires containing nickel ions [64]. In theory, a similar phenomenon may occur in the case of prosthetic restorations made of this metal. The corrosion of this alloy is subject to favourable conditions that may create ideal conditions for the development of a biofilm initiated by *Streptococcus* spp. (Increased acid secretion by *Streptococcus* spp. increases the progress of corrosion, but also increases the degree of growth of a given bacterial population).

C. Limitations and conclusions

The inflammatory effect of nickel ions and their connection with periodontal tissues is a slightly more complex issue. The increase in the production of hydroxyl ions will contribute to the initiation of a cascade of oxidation reactions and electron transfers that cause inflammation, but it is not of bacterial origin [64,71]. The released metal ions also cause cytotoxic effects by affecting periodontal fibroblasts and neoepithelial cells [13]. Perhaps, indeed, the effects on the periodontium may not be related to changes in the surrounding microbiome. This assertion can be substantiated by the findings of research undertaken by Daguci et al. The objective of the study was to evaluate the response of soft tissues to long-term exposure to fixed prosthetic devices composed of a nickel–chromium alloy. The results of the study indicated that Ni ions, released from the melt, induce gingival hyperplasia and chronic inflammation of the surrounding tissues. Furthermore, an increase in the number of CD3 T-cells was observed in immunohistochemistry studies [72].

The mechanisms of Ni-Cr alloys, like those of Ag-Pd alloys, centre primarily on a single element. Nickel is potentially toxic to cells and can also induce apoptosis and oxidative stress in oral cells [73]. Furthermore, this cytotoxicity is higher than in the case of Co-Cr alloys [53]. A significant issue would seem to be, for example, the interaction of Ni with the host’s immune system in the context of the oral microbiome. Unfortunately, there is a knowledge gap in this area.

#### 3.3.5. Titanium Alloys

A. Material characteristics.

Titanium and its alloys have become the materials of choice for modern dental prosthetics. The material’s functionality can be attributed to a number of factors, including its mechanical strength, biocompatibility, and corrosion resistance. The material’s capacity to stimulate integration with tissues, a property that is particularly relevant in implantology, is also worthy of note. Furthermore, the material’s relatively low density, a consequence of its lightness, is a desirable quality in dental prosthetics. The substance’s high reactivity necessitates the creation of specific environments for its procurement and that of its alloys [74]. The production of alloys is accomplished through various methods, including vacuum arc remelting (VAR), plasma arc melting (PAM), induction melting, and powder metallurgy. It is evident that two primary crystal structures of titanium are recognised: alpha and beta. The alpha phase is distinguished by a hexagonal structure that is characterised by high packing density. In contrast, the beta phase exhibits a cubic structure. These substances are both employed within the domain of dentistry [74].

B. Correlation with the oral microbiome.

⮚ Direct evidence

The study by Diaz et al. demonstrated that the presence of *S. mutans* in the environment promotes titanium corrosion [75]. A similar correlation was observed in a study by Chang et al., which utilised the Stern-Geary corrosion test, among other methods, to show that corrosion will be increased when a higher concentration of S. mutans is present. As posited by Chang et al., the inherent properties of titanium may impede the adhesion process of certain *Streptococcus* spp. [8]. However, under suboptimal conditions, the potential for corrosion remains, which could initiate further alterations in the microbiome [11]. The products of such corrosion may cause, for example, in the case of an implant, bone resorption and also act as a secondary inflammatory stimulus. Daubert et al. conducted a study involving 96 patients in order to ascertain how the microbiome changes when titanium is used in implant dentistry. The microbial microbiome was subsequently determined using 16S rRNA sequencing, and the dissolved titanium was measured. The study demonstrated that titanium has the capacity to modify the microbiological environment. In the case of the healthy implant area, a relatively large amount of *Prevotella* and *Haemophilus* was observed (contradicting previously reported studies that reported a reduction in *Prevotella*), with a simultaneous lower dissolution of titanium. In contrast, the prevalence of dissolved titanium, *Veillonella* spp., and *Streptococcus* spp. exhibited a marked increase in cases of *peri-implantitis*, suggesting a distinct microbial profile in this condition [11].

⮚ Circumstantial evidence

The correlation between titanium and bacteria remains to be fully elucidated. It has been determined that TiO_2_ exhibits anti-adhesive properties towards *S. sanguinis* and *S. mutans* [48,76]. Given the cariogenic properties of these bacteria, this suggests that limiting their adhesion may impede their multiplication, which could consequently reduce the risk of caries while maintaining other potentially optimal factors, such as proper oral hygiene [48]. A study by Liu et al. demonstrated that Ti-Cu alloys exhibit significant antimicrobial properties, notably reducing the survival and biofilm formation of *P. gingivalis* compared to pure titanium [77]. This effect is primarily attributed to the sustained release of copper ions, which exert a direct inhibitory effect on key periodontal pathogens. Given that *P. gingivalis* is a keystone pathogen responsible for initiating inflammatory processes in peri-implant tissues, the application of such bioactive alloys may contribute to a more favourable local environment [76]. However, the clinical impact of these materials remains a subject of discussion, as even titanium-based surfaces are susceptible to biocorrosion, which can further alter the peri-implant microbiome structure.

C. Limitations and conclusions

The relationship between the titanium base and the oral microbiome is not yet fully understood. The high biocompatibility is ensured by the previously mentioned TiO_2_ layer, which acts as a barrier against various chemical reactions [9]. Titanium has no antibacterial properties, which is why bacteria can colonise its surfaces [9]. However, there is no information on the effect of the titanium nanostructure itself and its alloy on the composition of the biofilm, nor on whether a “specific titanium microbiome” exists.

A summary of the above information is presented in Table 1.

### 3.4. Non-Metallic and Modern Aesthetic Materials

Although this publication focuses on metal alloys, it is worth mentioning a few words about ceramics. In general, ceramics are characterised by high aesthetic appeal, biocompatibility, and durability. The term “dental ceramics” is a general term for a fairly complex group of ceramic materials. The nomenclature includes several classifications of ceramics, for example, based on melting point or chemical composition [77].

As with metal alloys, proper processing of ceramics is essential. In the study by Kim et al., four ceramic materials were prepared, and intraoral clinical procedures were simulated. The samples were analysed using SEM imaging. The strain primarily studied was *S. gordonii*. The conclusions noted that intraoral polishing may result in greater biofilm accumulation. It is worth noting, however, that the study was conducted in vitro, which may impose limitations [78]. Yoon et al., on the other hand, conducted a study on humans also in the context of ceramics. In addition to ceramics, the formation of natural biofilm was compared on other materials, such as composite, metal alloys, and natural enamel. Discs were fabricated from the materials listed above, and patients then wore them in their mouths for a specified period of time. Based on SEM analysis, it was determined that surface roughness may influence biofilm formation [79]. An important ceramic-related material is VITA Enamic, a polymer-infiltrated ceramic for which relatively little microbiological information is available. It is currently known that VITA Enamic exhibits lower bacterial adhesion than some other CAD/CAM composites [80].

Polyaryletherketone (PAEK) materials, which include polyetheretherketone (PEEK) and polyetheretherketoneketone (PEKK) polymers, play an important role in dental materials science. PEEK is characterised by its excellent physical and chemical properties. PEKK, on the other hand, is a semi-crystalline polymer that is used in additive manufacturing processes [81]. The impact of these materials on the oral microbiome is not yet fully understood. One relevant study is that by Hung et al., who compared the bacterial colonisation of titanium and PEEK. This in vitro study evaluated the accumulation of Candida albicans biofilms. The results indicated that both materials exhibited similar bacterial adhesion; however, PEEK promoted biofilm growth with a lower amount of *T. denticola* [82].

### 3.5. Clinical Implications and Future Scope

The interaction between dental alloys and the oral microbiome is of significant importance in the clinical decision-making process, especially when planning treatment tailored to the patient’s individual needs. For example, in patients at high risk for dental caries or with a history of aggressive periodontitis, high-precious alloys (gold-based) or silver–palladium alloys should be preferred due to their biofilm-inhibiting and antibacterial properties. Conversely, base metal alloys (especially nickel–chromium alloys) should be avoided in patients with a high susceptibility to caries or suspected metal allergies due to their potential to cause dysbiosis and tissue hyperplasia. The potential release of titanium ions as a result of biocorrosion is strongly associated with a shift toward a pathogenic microbiome in peri-implant tissue inflammation under specific external environmental conditions. Physicians should implement rigorous long-term supportive care (follow-up visits) for patients with implants to monitor symptoms of inflammation [11,46,47,48,52].

There has indeed been progress in science and knowledge in this area. Although the current literature provides valuable baseline data, there are still significant gaps in our understanding that need to be addressed in future research: The shift toward dynamic models of multispecies biofilms: Most in vitro studies to date have focused on single-species biofilms (e.g., *Streptococcus mutans*). Future studies should utilise complex, multi-species biofilm models that accurately reflect the interactions between different members of the oral microbiome (e.g., between *Actinomyces viscosus* and *Candida albicans*). Furthermore, there is a serious shortage of long-term, prospective clinical studies evaluating how dental treatments modify the oral microbiome over the course of years, rather than weeks. High-throughput 16S rRNA sequencing should be widely implemented in clinical cohorts to capture subtle microbiological changes [4,11,69,70,77].

### 3.6. Limitations of the Study

Some of these limitations are included in Table 1. A significant issue with the presented studies is their reliance on in vitro methodologies. While these studies provide valuable mechanistic insights into ion release and initial cell adhesion, they cannot fully replicate the complex, dynamic and buffered environment of the human oral cavity. Many studies focus on single-species models; existing microbiological evaluations of dental alloys largely focus on isolated single-species cultures (primarily *Streptococcus mutans* or *Porphyromonas gingivalis*). This narrow approach fails to capture the complex, synergistic interactions that occur in mature, multi-species oral biofilms. The studies also feature short observation periods, consequently revealing a lack of data on the long-term impact of chronic biocorrosion and continuous low-dose metal ion release on the oral microbiome over several years. The small clinical cohorts are also significant. The few available in vivo and clinical studies are often limited by small sample sizes and high variability among patients in terms of baseline oral hygiene, diet and general health, which restricts the ability to generalise their results directly [4,6,11,12,46,47,48,58,60,66].

### 3.7. Discussion

Oral health is maintained by a balance between commensal and pathogenic species. Dominant bacterial groups, such as Bacillota (formerly Firmicutes), Actinomycetota (formerly Actinobacteria), and Pseudomonadota (formerly Proteobacteria), play a key role in this process. Prosthetic materials introduced into the oral cavity can disrupt this balance, contributing to biofilm formation, corrosion, or the release of metal ions. Compared to a healthy oral cavity, where the diversity of the microbiome remains relatively stable, the presence of alloys can disrupt this balance. Evidence from studies suggests that organic acids produced in the oral environment may accelerate the biocorrosion of certain base metal alloys, which in turn may create a local microenvironment conducive to the growth of acid-loving and cariogenic bacteria, whereas gold or titanium alloys have little effect on the microbial composition.

**Figure 2:**
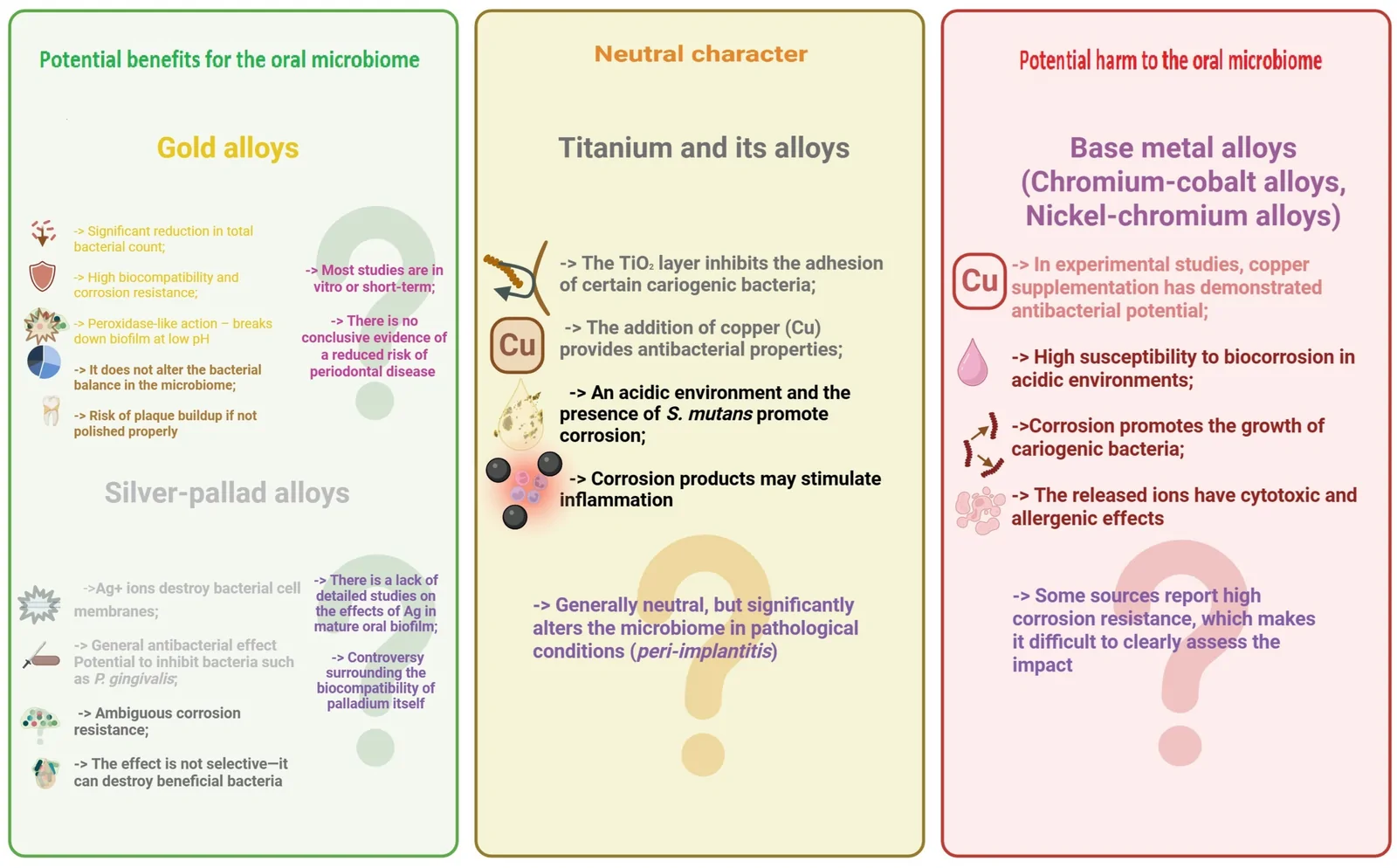
Classification of the described alloys based on their effect on the oral microbiome, with explanations. Created with BioRender.com.

However, these observations should be interpreted with caution due to the limited scope and high heterogeneity of the available literature. The clinical significance of these findings remains limited, as most studies are conducted in vitro, which does not fully reflect the complexity of the dynamic oral environment (such as saliva flow and interactions among multi-species biofilms). Furthermore, existing reports from in vivo studies are often limited by small sample sizes, a narrow taxonomic approach focusing exclusively on the most significant microbial components, and short observation periods. A summary of all this information is illustrated in Figure 2.

### 3.8. Conclusions

This review presents clear, material-specific findings regarding the impact of dental alloys on the oral microbiome. High-purity and precious alloys (gold, Ag-Pd) exhibit the most favourable and clinically cost-effective properties. High-purity gold alloys reduce the total bacterial count by up to 70% while maintaining the proportion of microorganisms. In contrast, silver and palladium alloys exhibit a strong, non-selective antibacterial effect against cariogenic and periodontal pathogens by damaging cell membranes. Titanium and Ti-Cu alloys generally exhibit high biocompatibility and a neutral nature thanks to a protective passivation layer. However, in pathological environments, increased dissolution of titanium is strongly correlated with a shift toward an aggressive, pathogenic microbiome. Copper-doped titanium (Ti-Cu) significantly inhibits biofilm formation by *P. gingivalis*. Base metal alloys (Co-Cr, Ni-Cr) have the least favourable effect and are associated with the highest risk of dysbiosis. Acid production by microorganisms destabilises their passive layers, causing severe biocorrosion and pitting. The released nickel ions exhibit high cytotoxicity and induce clinical tissue hyperplasia, while the cells formed as a result of corrosion actively promote the proliferation of cariogenic *Streptococcus* spp.

## Figures and Tables

**Table 1:** Comparison of the studies cited in the manuscript with their key findings.

Material	Study	Type of Study	Microorganisms Analysed	Key Findings	Limitations
Gold alloy	[46]	In vivo	Mixed microbiome, dental plaque	Significantly reduced amount of biofilm	No microbiological analysis, short observation period
	[47]	In vitro	*Streptococcus mutans*, *Streptococcus sanguis*, *Actinomyces naeslundii*, *Actinomyces viscosus*, *Porphyromonas gingivalis*, *Bacteroidetes* genus	The smooth surface of gold (after polishing) reduces the risk of bacterial buildup	Does not fully replicate the conditions of the mouth
Ag-Pd alloys	[48]	Review	*S. mutans*, oral pathogens	ROS production, antibacterial effect	Limited data
	[60]	Review	*Streptococcus mutans*, *Lactobacillus* spp., *Actinomyces* spp., *Bifidobacterium* spp., *Veillonella* spp., *Prevotella* spp., *Porphyromonas* spp.	Silver (particularly SDF and AgNPs) exhibits strong antimicrobial activity against cariogenic bacteria.	Lack of standardisation regarding the concentration and size of silver nanoparticles
	[58]	In vitro (Experimental)	Effects on cells	Ion release and low cytotoxicity of silver in the range of 55–243 μg/L	Mostly experimental
Co-Cr alloys	[12]	In vitro	No specific bacteria	Acidic pH increases corrosion	Artificial environment
	[66]	In vitro	No specific bacteria		
	[4]	In vitro	*S. aureus*, *E. coli*	CoCrCu has strong antibacterial properties thanks to the copper	No clinical trials
Ni-Cr, Ti	[8]	In vitro	*S. mutans*	Higher bacteria → increased corrosion	Contradictory evidence
	[69]	In vitro	For example, *Actinomyces viscosus*	Bacteria induce corrosion	No in vivo data
	[72]	Observational clinical study	Microorganisms were not analysed	Long-term use of old bridges made of Ni–Cr alloys and copper alloys was associated with a higher incidence of periodontal inflammation.	No measurements of metal ion concentrations in saliva or tissues were performed. No microbiological analysis of dental plaque was conducted.
Titanium and Ti-Cu alloys	[11]	Clinical	Mixed microbiome (*Veillonella*, *Prevotella*, *Streptococcus*)	Higher concentrations of titanium in the subgingival plaque were strongly associated with the presence of *peri-implantitis*.	Small group size
	[77]	In vitro (Experimental)	*P. gingivalis*, *S. aureus*, *E. coli*	Reduction in periodontal pathogens due to the presence of copper	Laboratory conditions
	[6]	In vitro	*S. mutans*	The bacteria lower the electrochemical potential of titanium, reducing its corrosion resistance; however, a passive layer still forms	Laboratory conditions

## Data Availability

No new data were created or analyzed in this study. Data sharing is not applicable to this article.
